# *ATG5* gene regulates testosterone synthesis of testicular Leydig cells in Hezuo pig

**DOI:** 10.3389/fvets.2025.1611919

**Published:** 2025-10-10

**Authors:** Hong Du, Zunqiang Yan, Haixia Shi, Shuangbao Gun

**Affiliations:** ^1^College of Animal Science and Technology, Gansu Agricultural University, Lanzhou, Gansu, China; ^2^Department of Reproductive Medicine, Lanzhou University Second Hospital, Lanzhou, Gansu, China; ^3^Gansu Research Center for Swine Production Engineering and Technology, Lanzhou, Gansu, China

**Keywords:** *ATG5*, autophagy, testosterone, Leydig cells, Hezuo pig

## Abstract

Autophagy-related gene 5 (*ATG5*) plays a crucial role in autophagosome formation. Recent studies have investigated the role of autophagy in regulating testosterone production; however, its expression in testicular tissues and Leydig cells of Hezuo pig remains less understood. In this study, we cloned the coding sequence (CDS) region of the *ATG5* gene and assessed its expression using qPCR across various tissues and testes at different developmental stages in Hezuo pigs. Subsequently, we constructed silencing and overexpression vectors for *ATG5* and transfected them into Leydig cells. Cell proliferation and apoptosis were evaluated using CCK-8 and flow cytometry assays, respectively. Autophagy and testosterone synthesized gene expression were detected by qPCR, while ATG5, StAR and LC3 protein levels were measured by Western blotting. Furthermore, testosterone concentration and the levels of autophagy-related genes BECN1 (Beclin1), NPC1L1 (Niemann-Pick C1-like 1), and TSPO (translocator protein) were quantified via ELISA. The results indicated that the CDS region of the *ATG5* gene spans 828 base pairs, encoding 275 amino acids. *ATG5* showed high expression in the testis and lung (*p* < 0.01), with significantly higher expression in the testicular tissues of the 4-month-old group compared to the 1-month-old group (*p* < 0.01). Compared to the empty vector control (ATG5-PC), the overexpression group (ATG5-OE) exhibited increased cell proliferation (*p* < 0.05), reduced apoptosis (*p* < 0.01), elevated autophagy gene expression (*BECN1, ATG7,* and *LC3*) (*p* < 0.01) and testosterone synthesis-related genes (*StAR, HSD3B* and *CYP11A1*) (*p* < 0.01), along with increased ATG5, StAR and LC3protein levels (*p* < 0.01), as well as significantly increased testosterone levels, BECN1, NPC1L1, and TSPO expression (all *p* < 0.01). These findings indicate that *ATG5* manipulation affects testosterone synthesis in Leydig cells, likely through autophagy regulation. This study offers novel insights into the function of *ATG5* in testosterone production within testicular Leydig cells, providing theoretical support for investigating early puberty in Hezuo pigs.

## Graphical abstract

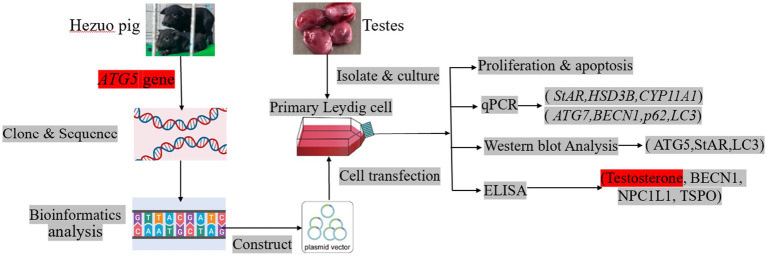

## 1 Introduction

Autophagy is a fundamental cellular process in eukaryotic cells, involving a metabolic pathway that transports damaged or dysfunctional intracellular components to the lysosome for degradation and recycling. This process is essential for maintaining normal cellular functions (1). Autophagy plays a role in various biological processes, such as mammalian follicular development (2), regulation of circadian rhythms (3), germ cell development (4), synthesis of steroid hormones (e.g., testosterone (5, 6), estrogen (7), progesterone (8, 9)), and maintenance of pregnancy (10). Autophagy is regulated by numerous conserved autophagy-related genes (11). In 1997, the first autophagy-related gene, *ATG1*, was cloned by A. Matsuura et al., and it plays a key role in initiating autophagy (12). To date, approximately 40 autophagy-related genes have been identified (13). Among these genes, *ATG5,* which is widely expressed in eukaryotes, regulates the autophagic ubiquitination process and plays a crucial role in the early stages of autophagosome formation and the initiation of autophagy (14). ATG5 initially forms an ATG5-ATG12 complex with ATG12, which subsequently interacts with ATG16 to form a homodimer. The ATG5-ATG12-ATG16 complex promotes the elongation and expansion of the autophagosome membrane, as well as the activation of autophagy (15–17).

Hezuo pig, a small plateau breed, is indigenous to the Gannan Tibetan Autonomous Prefecture in Gansu Province, China. This pig usually feeds on Juemas in the grasslands, and is also known as the Juema pig (18). Hezuo pig production is widespread in areas such as Hezuo, Xiahe, Luqu, Lintan, Zhuoni, and Diebu within the Gannan Tibetan Autonomous Prefecture, which has a cold climate and belongs to the alpine hilly regions characteristized by semi-agricultural and semi-pastoral activities (19). Hezuo pigs are small but robust, with high roughage digestibility and are highly adaptable due to prolonged rearing on grasslands. Compared to commercially introduced pig breeds (e.g., Yorkshire, Landrace, etc.), the Hezuo pig is noted for its early sexual maturity and stable inheritance. Boars display sexual desire at 45 days of age and typically reach sexual maturity around 4 months (20, 21).

In male mammals, Leydig cells account for about 4% of the total number of testicular somatic cells. Leydig cells are capable of secreting steroid hormones; studies indicate that over 95% of testosterone in the body is produced by these cells (22, 23). Testosterone is one of the important sex hormones in male mammals and is involved in reproductive activities such as the regulation of sexual maturation, spermatogenesis, and the maintenance of secondary sex characteristics (24, 25).

BECN1 (also known as Beclin 1) is the first gene identified as being associated with autophagy in mammals. BECN1 is a crucial for the autophagosomes formation, acting as an autophagic switch. It facilitates the localization of other autophagy proteins to autophagic vesicles, thereby regulating the formation and maturation of mammalian autophagosomes (26–28). Microtubule-associated protein 1 light chain 3 (LC3) is one of the key proteins expressed during autophagy, playing a crucial role in the formation and maturation of autophagosomes. It is commonly utilized as a marker to monitor autophagic activity, with its expression level exhibiting a positive correlation with autophagic flux (29–31). Cholesterol serves as the substrate for testosterone synthesis. The steroidogenic acute regulatory protein (StAR) binds cholesterol in the outer mitochondrial membrane and transports it to the inner membrane, where it is catalyzed by the cytochrome P450 side-chain cleavage enzyme to produce pregnenolone. After leaving the mitochondria, pregnenolone is converted into testosterone in testicular tissue (32, 33). Transporter protein (TSPO), located on the outer mitochondrial membrane, is essential for the import of cholesterol into the inner mitochondrial membrane, representing the rate-limiting step in steroid hormones biosynthesis (34, 35). Recently studies show that Niemann-Pick C1-like 1 (NPC1L1) is a transmembrane cholesterol absorption transporter capable of mediating cholesterol uptake, regulating lipid homeostasis in mammals and increasing substrates for synthesizing steroid hormones, cholesterol depletion has been associated with autophagy (36, 37).

This study focused on the Hezuo pig, the *ATG5* gene was cloned and sequence analysis was performed. Subsequently, Leydig cells from Hezuo pig were isolated. Based on successful transfections with *ATG5* silencing and overexpression vector, the effects of *ATG5* overexpression and silencing on testosterone levels, BECN1, NPC1L1, TSPO were explored using ELISA assays; autophagy gene (*BECN1, ATG7, p62* and *LC3*) and testosterone synthesis gene (*StAR, HSD3B,* and *CYP11A1*) were detected by qPCR; ATG5, StAR and LC3 were detected by Western Blot, aiming to elucidate the molecular mechanisms by which *ATG5* regulates testosterone synthesis in testicular Leydig cells of Hezuo pigs, with the goal of investigating the reproductive endocrine basis underlying the characteristic precocious puberty trait in this breed.

## 2 Materials and methods

### 2.1 Ethical statement

The entire study was approved by the Institutional Animal Care and Use Committee of Gansu Agricultural University. All experimental procedures and sample collection methods adhered to the approved guidelines to ensure animal welfare.

### 2.2 Sample collection

A total of 6 1-month-old (1 M, *n* = 3) and four-month-old (4 M, *n* = 3) Hezuo pigs, raised by farmers in Gannan, Gansu, China, were selected. After slaughter, testicular, heart, liver, spleen, lung, and kidney tissues were collected, quickly frozen in liquid nitrogen, transported to the laboratory, and stored at −80°C for RNA extraction. Testicular tissues were collected from the center of the testis and immediately immersed in 3% glutaraldehyde for transmission electron microscopy. Testicular tissues from 1 M Hezuo pigs were sterilized by immersion in 75% alcohol for 3 min, then placed in pre-cooled PBS buffer containing 2% penicillin–streptomycin (Gibco, Carlsbad, CA, USA), and transported back to the laboratory within 2 h for the isolation of testicular Leydig cells.

### 2.3 Total RNA extraction and cDNA synthesis

Total RNA was extracted from each sample using TRIzol reagent (AG, Changsha, Hunan, China) following the manufacturer’s instructions. The concentration and quality of the RNA samples were assessed using a NanoDrop2000 spectrophotometer (Thermo Fisher Scientific, Waltham, MA, USA). The RNA was then reverse-transcribed into cDNA using the Evo M-MLV RT Kit (AG, Changsha, Hunan, China) and stored at −20°C.

### 2.4 Primer synthesis

Based on the porcine gene sequence in Genbank, primers were designed using primer 5.0 software (Premier Company, Toronto, ON, Canada), and synthesized by Genewiz Biotechnology Co. (Suzhou, Jiangsu, China). The primer details are presented in Table 1.

**Table 1:** A list of the primers used in the study.

Gene name	Transcript no.	Primer sequences (5′–3′)	Length (bp)
*ATG5*-1	NM_001037152.2	F: GGTTGTCTTGGCTGGATA R: ACTGAAGCAGAAGGGTGA	1,172
*ATG5*-2		F: CACTGCCGTCATTCAACT R: CCAATGTTTCCACTCCCT	182
*GADPH*	NC_058085.1	F: AGCAATGCCTCCTGTACCAC R: AAGCAGGGATGATGTTCTGG	140
*StAR*	NM_213755.2	F: TTCGACGTCGGAGCTCTCT R: CTTTACTCAGCACCTCGTCCC	118
*CYP11A1*	NM_214427.1	F: CAGGCTGAATGTTTGGTTTGGAAGAAG R: AGGAGGAGGAGAGGAGGAAGTAGG	124
*HSD3B*	NM_001004049.2	F: GGTCTTCATCCACACCAGCA R: GCTCCCCTCCCCGTAGATAT	121
*p62*	XM_003123639.4	F: CCGTCTACAGGTGAACTCCAGTC R: GGTACAATGCCGCTTCCTTCAG	109
*BECN1*	NM_001044530.1	F: GCTGCCGTTGTACTGTTCTGG R: GTCTCGCCTTTCTCAACCTCTTC	120
*ATG7*	NM_001190285.1	F: TGGTCATCAATGCTGCGTTGG R: TCACAGGGTTGCTGGGACAC	108
*LC3*	NM_001190290.1	F: TCATCCGAGAGCAGCATCCTAC R: ATGTTGACATGATCAGGCACCAG	115
*Bcl2*	XM_012103831.3	F: CGCAGAGGGGCTACGAGTG R: CGGGCTGGGAGGAGAAGATG	90
*PCNA*	NM_001291925.1	F: AGAGGAGGAAGCAGTTACCATAGAG R: ACTGAGTGTGACTGTAGGAGAGAG	115
*Caspase3*	XM_015104559.2	F: TGGGATTGAGACGGACAGTGG R: TCGCCAGGAATAGTAACCAGGTG	112
*Caspase9*	NM_001277932.2	F: TGCCCACACCTAGTGACATCTTG R: TGCTCCAGAACGCCATCCAG	115

### 2.5 PCR amplification

The cDNA samples derived from the testicular tissues of all Hezuo pigs were used as a templates to amplify the CDS sequence of the *ATG5* gene. The PCR reaction system (20 μL) contained of: 2.0 μL cDNA, 1.0 μL forward primer, 1.0 μL reverse primer, 10 μL Easy Taq PCR SuperMix (Tiangen Biotech, Beijing, China), and 6 μL of RNase free H_2_O. The reaction conditions were as follows: initial denaturation at 95°C for 5 min; 35 cycles of denaturation at 94°C for 30 s, annealing at 59°C for 30 s, and extension at 72°C for 80 s; followed by a final extension at 72°C for 10 min.

### 2.6 Cloning and sequencing

The PCR product of the Hezuo pig *ATG5* gene was purified using 1.5% agarose gel electrophoresis, then ligated into the pMD19-T vector (TaKaRa, Dalian, Liaoning, China) and transformed into DH5α competent cells (TransGen Biotech, Beijing, China). Cells were plated on LB solid medium without ampicillin (AMP^+^), X-Gal, and IPTG, and incubated overnight at 37°C. Independent positive clones were selected and inoculated into 5 mL of LB liquid medium containing ampicillin (AMP+) and incubated with shaking for 12–16 h. A 2 μL aliquot of the bacterial culture was used for PCR verification, while the remaining culture was used for plasmid DNA extraction and subsequent sequencing by Shenggong Biotech Co., Ltd. (Shanghai, China).

### 2.7 ATG5 expression assay

The expression of *ATG5* in various tissues and testicular tissues of Hezuo pigs at different ages was detected using qPCR on the Roche LightCycler 96 system (Roche, Basel, Switzerland). The reaction mixture was 20 μL, containing: 10 μL SYBR Premix Ex Taq II, 1.0 μL forward primer, 1.0 μL reverse primer, 2.0 μL cDNA, and 6 μL RNase-free water. The thermal cycling protocol included an initial denaturation at 95°C for 3 min, followed by 40 cycles of denaturation at 95°C for 15 s, annealing at 58°C for 15 s, and extension at 72°C for 20 s. All reactions were carried out in three technical replicates. The relative expression of all reactions were performed in triplicate. The relative expression level of *ATG5* was calculated relative to GAPDH as the reference gene using 2^–ΔΔCt^ method (38).

### 2.8 Bioinformatics analysis

The CDS region of the *ATG5* gene obtained by cloning was analyzed using various online tools and databases. Homologous sequences were identified using the BLAST tool on the NCBI website. Details of the specific software and websites used are provided in Table 2.

**Table 2:** A list of websites and software of bioinformatics analysis in the study.

Software	Websites	Function
BLAST	http://blast.ncbi.nlm.nih.gov/Blast.cgi	Sequence alignment
MEGA 7.0	MEGA 7.0 software	Sequence alignment
ProtParam Server	https://web.expasy.org/protparam/	Physicochemical properties
Protscale	http://web.expasy.org/protscale/	Hydrophobicity
TMHMM-2.0 Server	http://www.cbs.dtu.dk/services/TMHMM-2.0/	Protein transmembrane domains
SWISS-MODEL	https://swissmodel.expasy.org/	Tertiary structures
SOPMA	https://npsa-prabi.ibcp.fr/cgi-bin/npsa_automat.pl?page=npsa_sopma.html	Secondary structures
STRING 11.0 database	https://string-db.org/cgi/input.pl?sessionId=uvjABp4Tn4Dw&input_page_show_search=on	Protein interactions

### 2.9 Isolation, purification and identification of Hezuo pig testicular Leydig cells

The white membrane on the surface of the testicular tissue was removed to expose the testicular parenchyma, which was then minced and transferred to a 50 mL centrifuge tube. Type IV collagenase, pre-warmed to 37°C and at a concentration of 1 mg/mL, was added for digestion at 37°C for 60 min. The supernatant was aspirated, diluted with an equal volume of PBS, and sequentially filtered through 70 μm and 40 μm cell strainers. The filtrate was collected by centrifugation at 1,100 rpm for 10 min. The pellet was collected and washed twice with PBS. Cells were resuspended in DMEM supplemented with 10% FBS (Gibco, NY, USA) and passed through 70 μm and 40 μm cell strainers. The filtrate was collected and inoculated into culture flasks. After 4 h, non-adherent cells were removed, followed by two washes with PBS. Cells were then re-suspended in DMEM containing 10% FBS and cultured in an incubator set at 37°C and 5% CO₂. Culture medium was replaced every 48 or 72 h based on cell growth (39).

The specific 3β-HSD antibody (Bioss Biotechnology Co., Ltd., Beijing, China) was used to identify Leydig cells by fluorescent immunostaining. The purified cells were seeded in 24-well plates and cultured until they reached approximately 70% confluence, then fixed with 4% paraformaldehyde for 15 min, permeabilized with 1% Triton X-100 (Beyotime Biotechnology, Shanghai, China) for 15 min, blocked it with 5% (w/v) goat serum for 30 min at room temperature. Next, the primary antibody (anti-HSD3B, 1: 500) was incubated overnight at 4°C. After washing with PBS (three times, 5 min each), the cells were incubated with secondary antibody (Cy5-labeled goat anti-rabbit IgG, 1:500) and a nuclear counterstain (4′,6-diamidino-2-phenylindole, DAPI) for 1 h at room temperature in the dark. Following three additional 5-min washes with distilled water, the samples were mounted and examined under a fluorescence microscope (Nikon, Eclipse C1, Tokyo, Japan).

### 2.10 *ATG5* gene silencing or overexpression vector construction and cell transfection

Based on the gene clone sequence, si-ATG5-404, si-ATG5-477, si-ATG5-566, and si-ATG5-696 interference sequences were designed. The negative control (si-ATG5-NC) served as the control group. The interference sequences are listed in Table 3. The *ATG5* overexpression vector (ATG5-OE) was constructed using the pcDNA 3.1 cloning vector, employing 5’ HindIII and 3’ BamHI restriction sites. The empty vector (ATG5-PC) served as a control. The silencing and overexpression vectors were synthesized by GenePharma Biotech Co., Ltd. (Shanghai, China).

**Table 3:** The information of interference RNA sequence.

Vector	Sense (5′–3′)	Antisense (5′–3′)
si-ATG5-NC	GGGAUGAGAAAGCCAUAAATT	UUUAUGGCUUUCUCAUCCCTT
si-ATG5-404	CCCUCUAUCAGGAUGAGAUTT	AUCUCAUCCUGAUAGAGGGTT
si-ATG5-477	GACGUUGGUAACUGACAAATT	UUUGUCAGUUACCAACGUCTT
si-ATG5-566	CACCACUGAAAUGGCAUUATT	UAAUGCCAUUUCAGUGGUGTT
si-ATG5-696	GGAUGUAAUUGAAGCUCAUTT	AUGAGCUUCAAUUACAUCCTT

Hezuo pig testicular Leydig cells were seeded into 6-well culture plates. Once cell confluence reached approximately 80%, the silencing and overexpression vectors were transfected into each well following the protocol of Lipofectamine 2000 Reagent (Invitrogen, Carlsbad, CA, USA). qPCR was performed to quantify the silencing or overexpression of *ATG5* mRNA in Leydig cells. Successful transfection was indicated by an mRNA expression level in the overexpression group exceeding 10-fold that of the PC group, and in the silencing group being less than 0.5-fold of the NC group.

### 2.11 Effects of ATG5 gene silencing or overexpression on proliferation and apoptosis of testicular Leydig cells

Hezuo pig testicular Leydig cells in the logarithmic growth phase were selected and seeded into 96-well plates at a density of 1 × 10^4^ cells/well. Transfection was performed 24 h post-seeding. At 24, 48, and 72 h post-transfection, 10 μL of CCK-8 reagent (Beyotime, Shanghai, China) was added to each well. Following a 2-h incubation at 37°C, the optical density (OD) at 450 nm was measured using a microplate reader, and the growth curves were plotted.

Apoptosis was assessed using flow cytometry. Cells were harvested using EDTA-free trypsin to create a single-cell suspension, washed with chilled PBS, and resuspended in 300 μL of binding buffer at a concentration of approximately 5 × 10^5^ cells/tube. Then, 5 μL of Annexin V-FITC and 5 μL of propidium iodide (PI) were added, gently mixed, and incubated in the dark for 10 min. The samples were analyzed by flow cytometry.

### 2.12 qPCR assay the effects of ATG5 gene silencing or overexpression on autophagy and testosterone synthesized gene expression of testicular Leydig cells

Hezuo pig Leydig cells were seeded in 6-well plates and transfected when they reached approximately 70% confluence. At 48 h post-transfection, the culture medium was removed, and the cells were washed three times with ice-cold PBS. Total RNA was extracted from each well’s Leydig cells using 1 mL of TRIzol reagent according to the protocol outlined in Section 4.3. The extracted RNA was then reverse-transcribed into cDNA. Expression levels of autophagy-related genes (*BECN1, p62, ATG7* and *LC3*) and steroidogenesis-related genes (*StAR, HSD3B* and *CYP11A1*) across different treatment groups were quantified using the reaction system specified in Section 4.7 and the amplification protocol detailed in Table 1.

### 2.13 Western blot analysis

Hezuo pig Leydig cells were seeded in culture flasks. When cell confluence reached approximately 80%, silencing and overexpression vectors were introduced into each flask. At 48 h post-transfection, total proteins were extracted using a radioimmunoprecipitation assay (RIPA) lysis buffer (Solarbio, Beijing, China) supplemented with phenylmethanesulfonyl fluoride (PMSF) (Solarbio, Beijing, China), following the manufacturer’s instructions. Protein concentrations were quantified using a bicinchoninic acid (BCA) protein assay kit (Beyotime, Shanghai, China) to standardize the sample volumes. A mixture of 30 μL loading buffer and 120 μL protein samples was transferred to a 1.5 mL centrifuge tube and denatured by boiling at 95°C for 15 min. The protein samples were separated by 12.5% sodium dodecyl sulfate-polyacrylamide gel electrophoresis (SDS-PAGE) and subsequently transferred onto polyvinylidene difluoride (PVDF) membranes (Beyotime, Shanghai, China). The membranes were blocked with 5% non-fat milk in phosphate-buffered saline with Tween-20 (PBST) for 1 h at room temperature and then incubated overnight at 4°C with rabbit anti-ATG5 and anti-StAR polyclonal antibodies (1:2,000; Bioss, Beijing, China) as well as GAPDH antibody (1:2,000; Bioss, Beijing, China). After washing three times with PBST, the PVDF membranes were incubated with horseradish peroxidase (HRP)-conjugated secondary antibody (Proteintech, Wuhan, Hubei, China; 1:10,000) at 37°C for 1 h. The positive signals of the target proteins were visualized using an enhanced chemil The positive signals of the target proteins were visualized using an enhanced chemiluminescence (ECL) kit (Servicebio, Wuhan, Hubei, China). Gray levels were analyzed using ImageJ2 software (National Institutes of Health, Bethesda, MD, USA). The relative expression levels of ATG5 and StAR proteins were normalized to those of GAPDH.

### 2.14 Enzyme-linked immunosorbent assay (ELISA) assay

Hezuo pig testicular Leydig cells were seeded in 6-well culture plates. When cell confluence reached approximately 80%, silencing and overexpression vectors were introduced into each well. After 48 h post-transfection, the supernatant from each well was collected to perform ELISAs using the Testosterone Assay Kit, Autophagy Gene Assay Kit, NPC1L1 Assay Kit, and TSPO Assay Kit (Nanjing Jingmei, Nanjing, China), following the manufacturers’ instructions.

### 2.15 Statistical analysis of data

All assays were repeated independently at least three times. SPSS26.0 software (SPSS, Chicago, IL, USA) was executed to analyze the data. Two-tailed student’s t-test was used for comparison between two groups, one-way ANOVA was used for comparison between more than two groups, Duncan’s method was used for multiple comparisons. The results were expressed as mean ± standard deviation (Mean ± SD), *: indicate significant differences (*p* < 0.05); **: indicate extremely significant differences (*p* < 0.01).

## 3 Results

### 3.1 Autophagosomes confirmed by TEM

**Figure 1:**
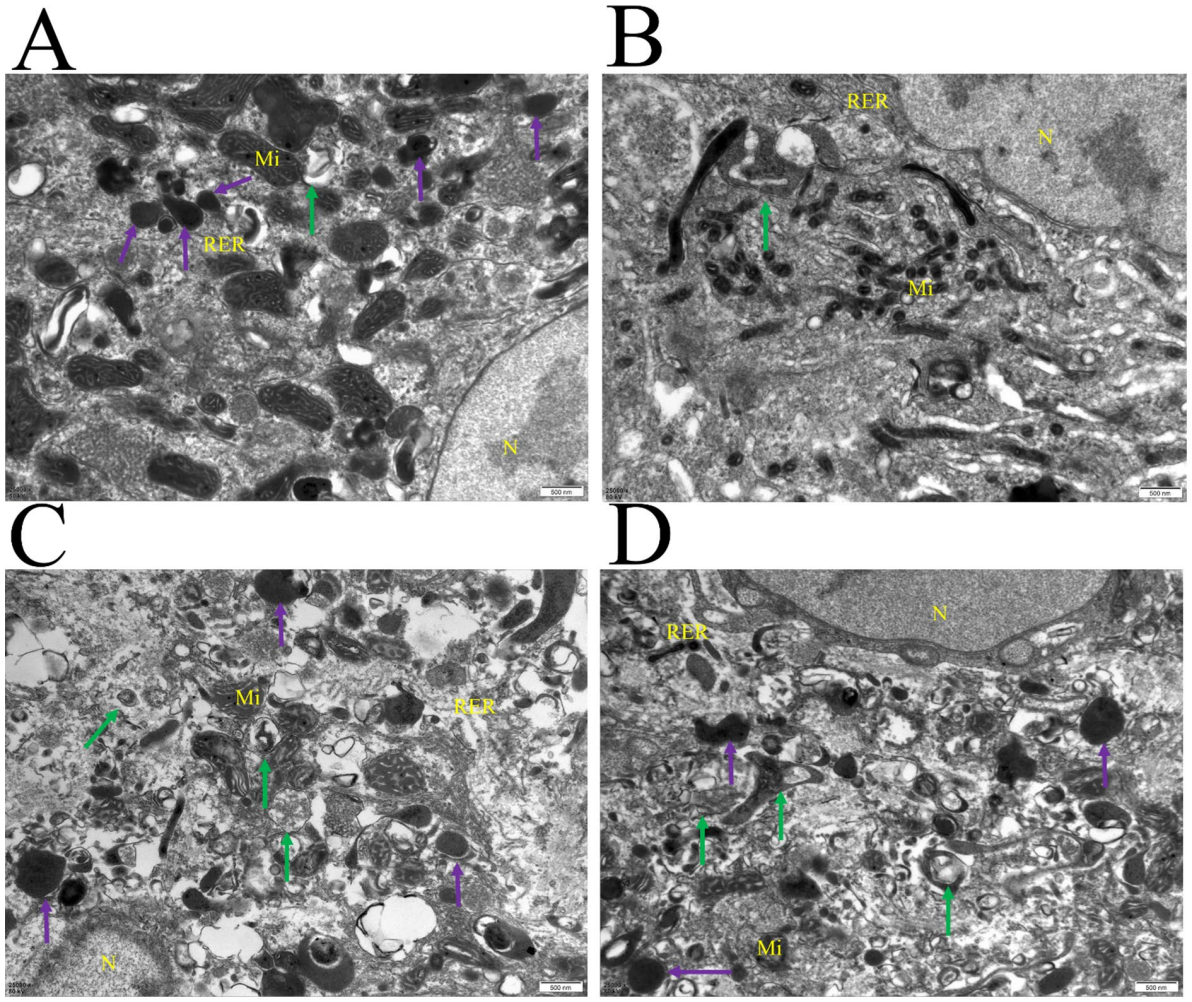
Autophagy was observed in the testicular tissues of Hezuo pig. Nucleus (N), mitochondria (Mi), rough endoplasmic reticulum (RER); autophagosome (green arrow), lysosome (purple arrow). Scale bar = 50 nm. **(A,B)** Represent samples from 1-month-old pigs, while **(C,D)** are from 4-month-old pigs.

Autophagy and lysosomes were confirmed through transmission electron microscopy (TEM). The autophagy-related structures and lysosomes, which typically have a double membrane, were easily detected in the testicular tissues of 1-month-old and 4-month-old Hezuo pigs (Figure 1). Once parts of the cytoplasm are sequestered within autophagosomes, their contents and surrounding membranes remain morphologically stable (Figure 1). Our findings confirm the occurrence of autophagy in testicular tissues of Hezuo pigs at both 1 month and 4 months, with autophagy being more pronounced at 4 months.

### 3.2 CDS sequence characterization of ATG5

**Figure 2:**
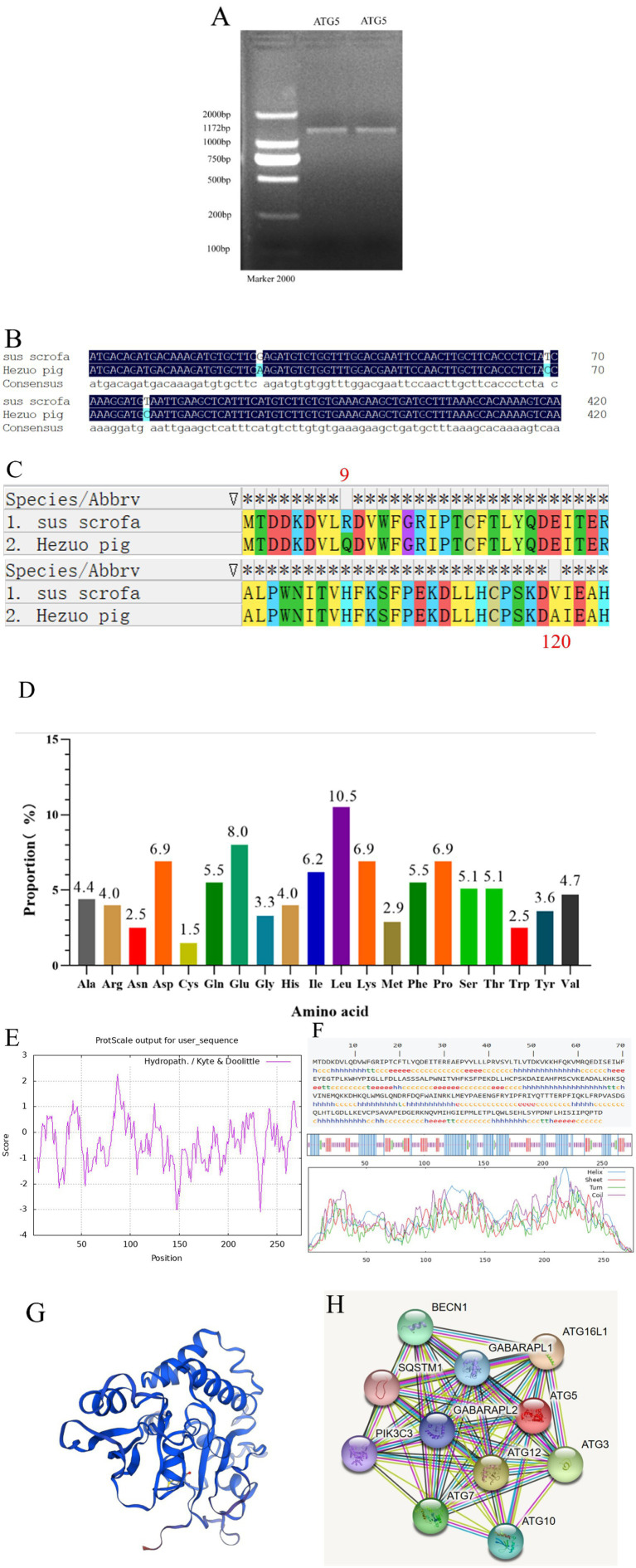
Cloning and sequence analysis of Hezuo pig *ATG5* gene. PCR amplification product of *ATG5* gene **(A)**; Sequence alignment between cloned and reference *ATG5* CDS region **(B)**; Amino acids sequence alignment between cloned and reference *ATG5* **(C)**; Analysis of the amino acid composition of Hezuo pig ATG5 protein **(D)**; The analysis of hydrophobicity of Hezuo pig ATG5 protein **(E)**; The secondary structure prediction of Hezuo pig ATG5 protein **(F)**; The transmembrane structure prediction of the Hezuo pig ATG5 protein **(G)**; Analysis of protein networks interacting of ATG5 protein **(H)**.

The PCR product of the *ATG5* gene was detected using 1.5% agarose gel electrophoresis, resulting in a specific band of approximately 1,172 bp (Figure 2A). Sequencing and BLAST comparison results revealed the coding sequence (CDS) region was 828 bp in length, encoding 275 amino acids. Three nucleotides were mutated (base 26 G → A, base 69 T → C, base 359 T → C) (Figure 2B). Two of these mutations resulted in missense mutations, while one was a synonymous mutation, leading to two amino acid changes (R → Q, 9st point, V → Q120st point) (Figure 2C). Among the 275 amino acids, leucine accounted for the largest proportion (10.5%) (Figure 2D) Phenylalanine at position 87 exhibited the strongest hydrophobicity (score: 2.267), while glutamic acid at 233 position showed the weakest hydrophobicity (score: −3.100), suggesting that the protein encoded by this gene is hydrophilic (Figure 2E). The predicted physicochemical properties of the ATG5 protein included a molecular formula of C_1477_H_2248_N_378_O_418_S_12_, a molecular weight of 32,373.10, a theoretical pI of 5.47, and an instability index (II) of 45.92. These results indicate that ATG5 is an unstable protein. The predicted secondary structure of the ATG5 protein revealed a mixed composition, consisting of 44% random coil, 36.36% alpha helix, 15.27% extended strand, and 4.36% beta turn (Figure 2F). The predicted tertiary structure of the ATG5 protein primarily consists of random coils, alpha helices, and beta turns (Figure 2G). The protein encoded by ATG5 may interact with 10 proteins, primarily including autophagy-related proteins such as ATG12, ATG16 and ATG3 (Figure 2H).

### 3.3 Expression pattern of ATG5 at the transcript levels in Hezuo pig

**Figure 3:**
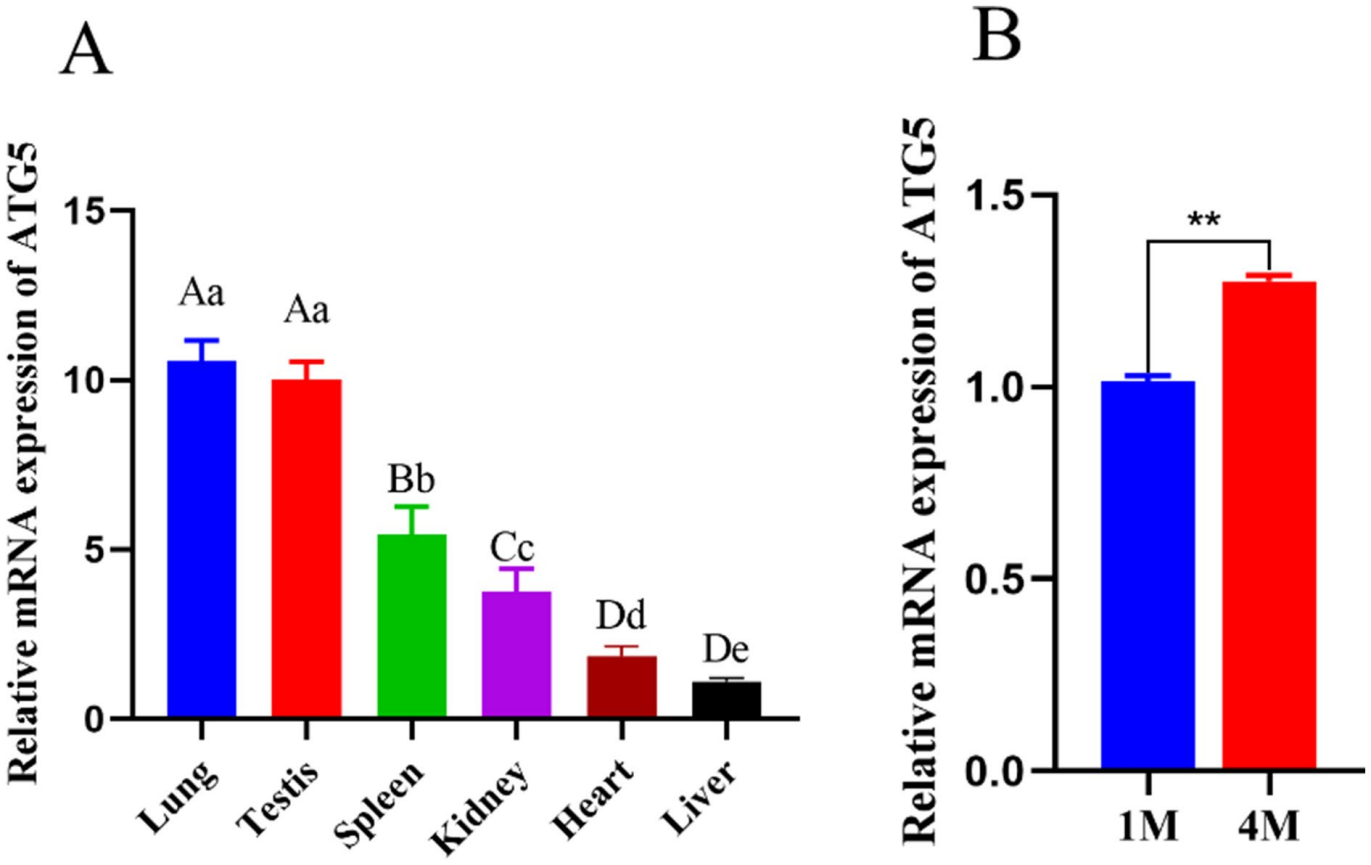
Expression of *ATG5* mRNA in different tissues of Hezuo pig **(A)**, Expression of *ATG5* in different month of Hezuo pig **(B)**. Different uppercase letters indicate extremely significant differences (*p* < 0.01), different lowercase letters indicate significant differences (*p* < 0.05).

qPCR analysis of *ATG5* mRNA expression across various tissues indicated higher expression levels in the testis and lung compared to those in the heart, spleen, kidney, and liver (Figure 3A). Within testicular tissues, *ATG5* mRNA was extremely significantly increased in the 4-month group compared to 1-month group (*p* < 0.01) (Figure 3B).

### 3.4 Purification and identification of Hezuo pig primary Leydig cells

**Figure 4:**
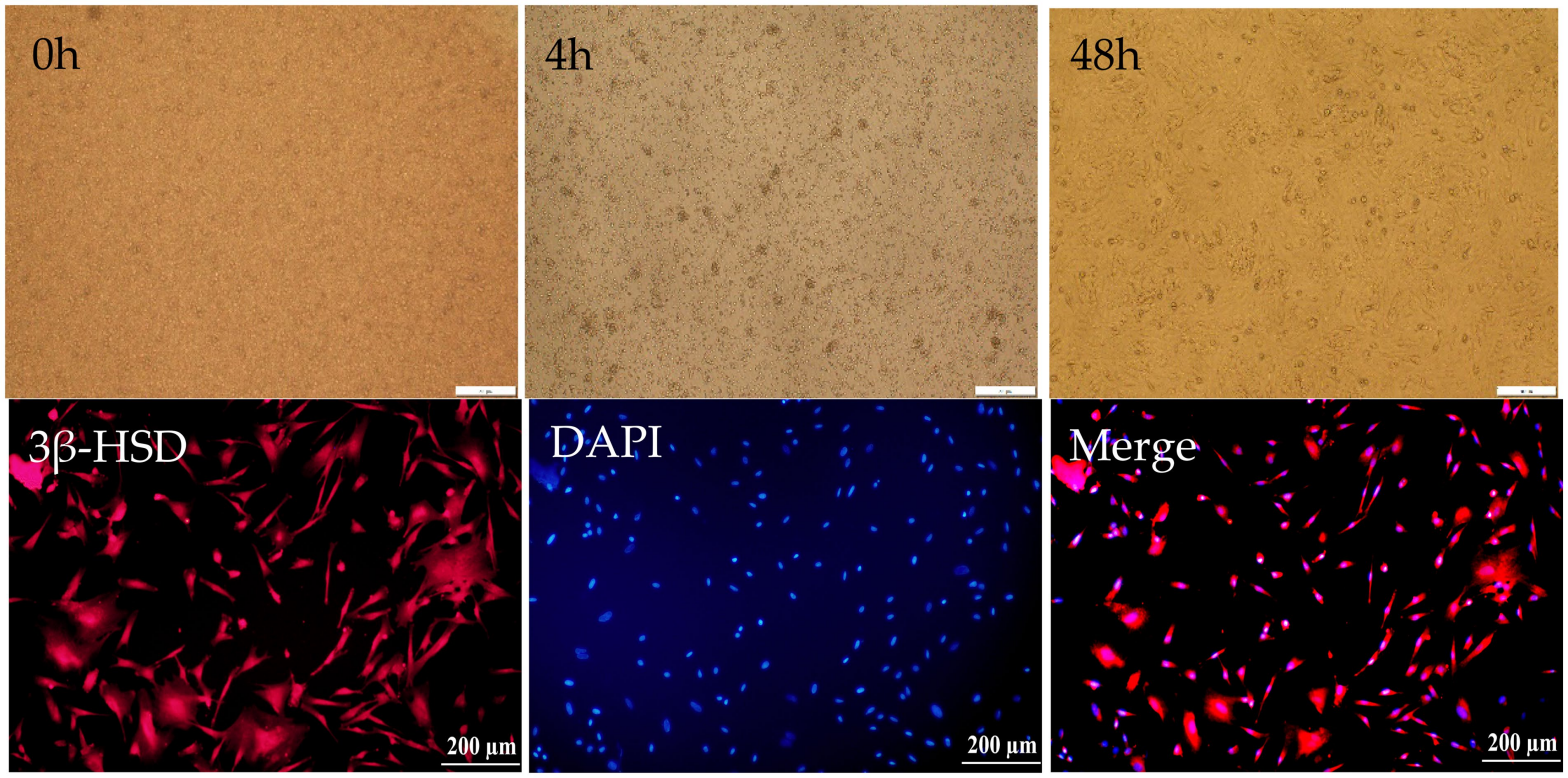
Isolation and identification of Leydig cells in Hezuo pig. Primary of Leydig cells in Hezuo pig just isolated (20×) (0 h), primary of Leydig cells in Hezuo pig after 4 h culturing (20×) (4 h), primary of Leydig cell in Hezuo pig after 48 h culturing (20×) (48 h). Immunofluorescence identification of 3β-HSD in Leydig cells of Hezuo pig (20×) (3β-HSD). DAPI:4′6-Diamidino-2-phenylindole in Leydig cells of Hezuo pig (20×) (DAPI). Merge of 3β-HSD and DAPI in Leydig cells of Hezuo pig. (20×) (Merge).

After being isolated from testicular tissue, Leydig cells appeared round or oval, displaying good refractive properties and remained suspended in the culture medium. To further purify the culture, the medium was changed 4 h post-isolation, allowing for the removal of unadhered cells. Non-adherent cells were subsequently eliminated through multiple medium changes. Leydig cells were identified using a specific 3β-HSD antibody and immunofluorescence. All cells exhibited positive staining, confirming that the isolated cells were indeed testicular Leydig cells (Figure 4).

### 3.5 Transfection efficiency assay

**Figure 5:**
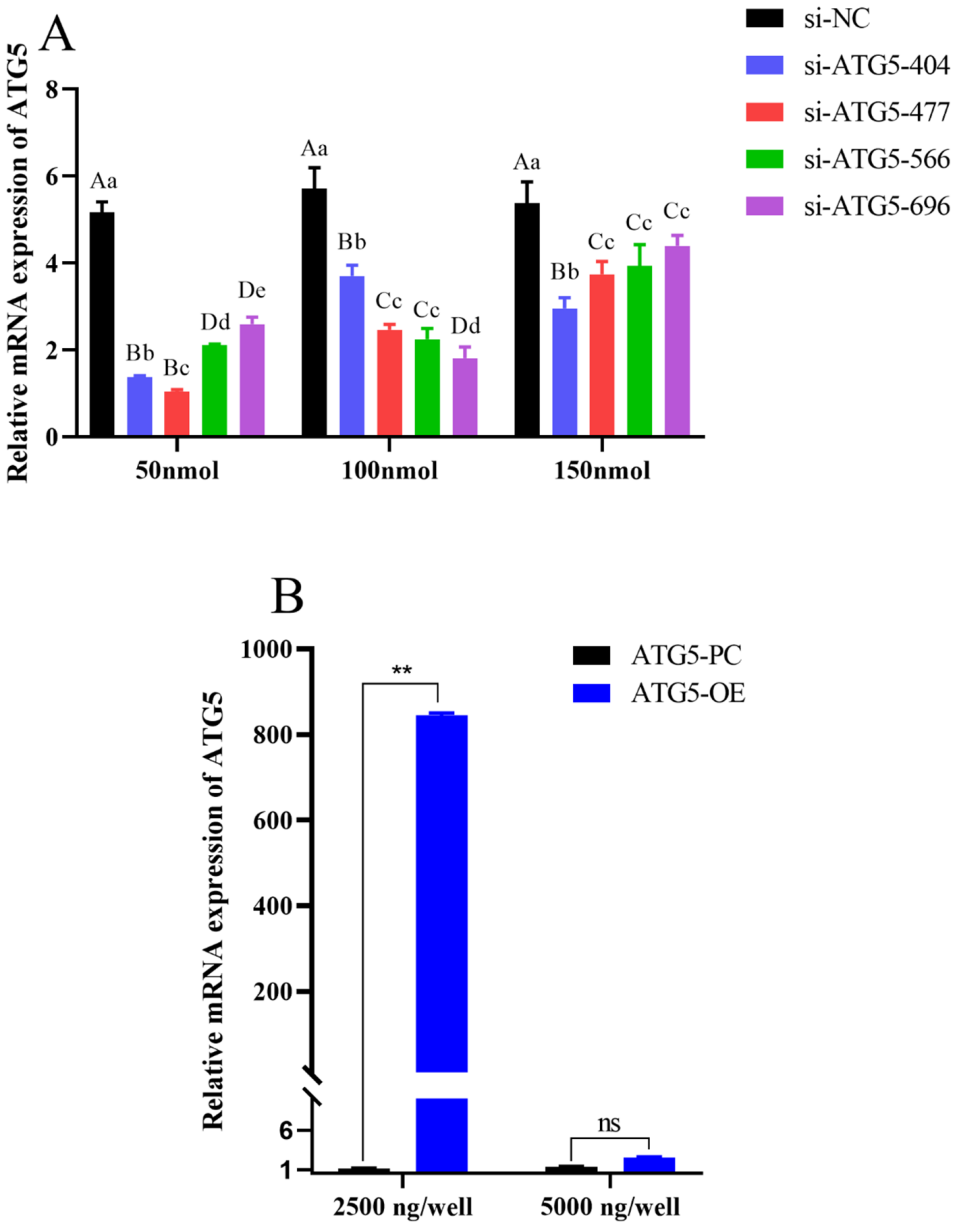
Detection of transfection efficiency. **(A)** *ATG5* gene silencing efficiency of different concentration. **(B)** *ATG5* gene overexpression efficiency of different concentration. Different uppercase letters indicate extremely significant differences (*p* < 0.01), different lowercase letters indicate significant differences (*p* < 0.05).

qPCR was used to detect the mRNA expression level of the *ATG5* gene in transfected Hezuo pig testicular Leydig cells. The results showed that the *ATG5* gene was significantly down-regulated in the si-ATG5-404, si-ATG5-477, si-ATG5-566, and si-ATG5-696 transfected groups compared with si-ATG5-NC at 48 h, in which the silencing effect of si-ATG5-477 was extremely significantly higher than the other three (*p* < 0.01) (Figure 5A). Conversely, the *ATG5* gene was up-regulated following transfection with a 2,500 ng concentration of the overexpressed ATG5-OE for 48 h (Figure 5B) (*p* < 0.01). The above results demonstrate both the interference and overexpression were successful.

### 3.6 Effect of ATG5 gene silencing or overexpression on the proliferation, apoptosis of Hezuo pig Leydig cells

**Figure 6:**
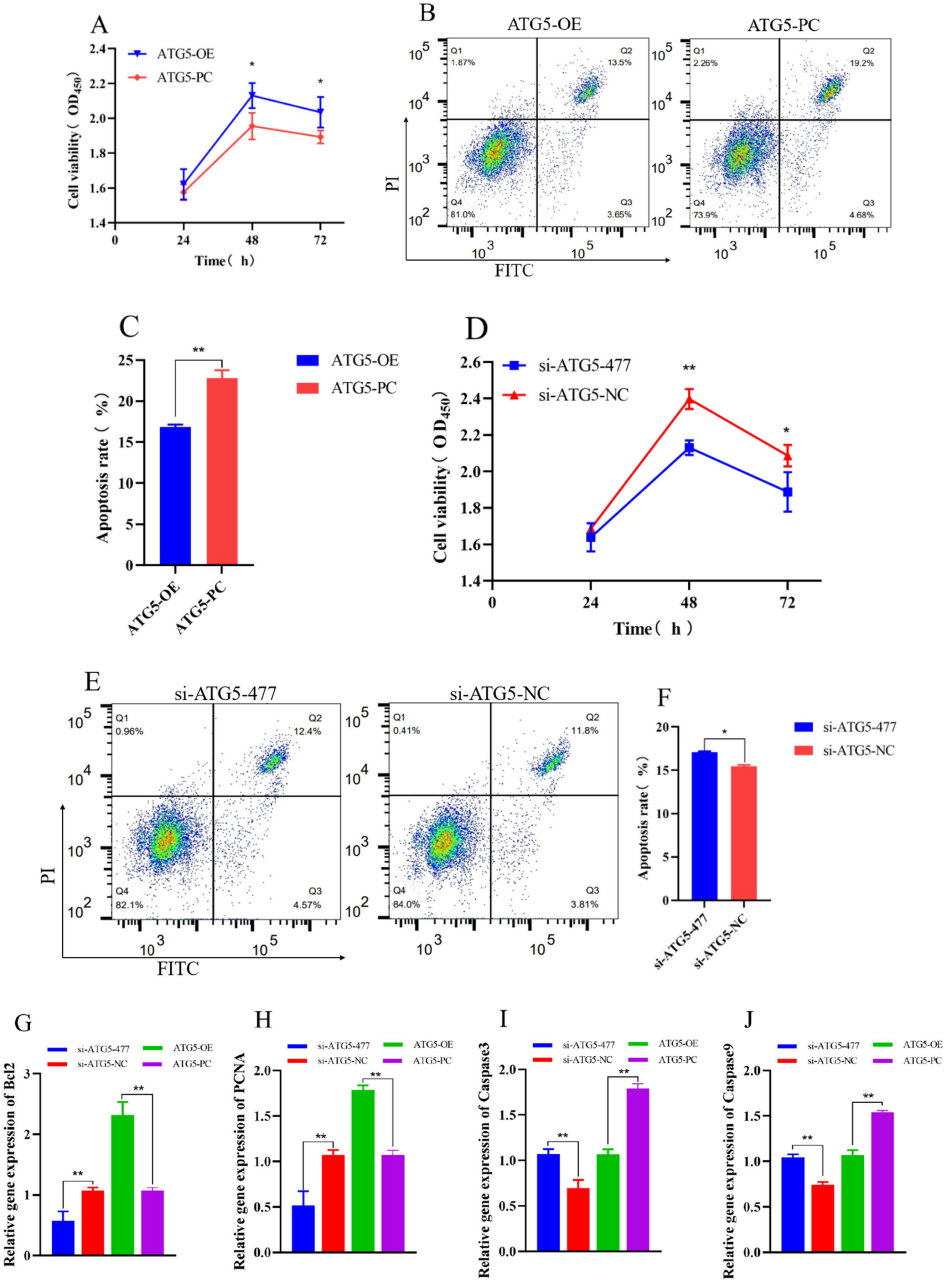
Effect of *ATG5* gene silencing or overexpression on the proliferation, apoptosis of Hezuo pig Leydig cells. CCK-8 detects cell proliferation rate after overexpression or silencing *ATG5* gene **(A,D)**, Flow cytometry detection cell apoptosis rate after overexpression or silencing *ATG5* gene **(B,C,E,F)**, qPCR detection relative gene expression of *Bcl2, PCNA, Caspase3* and *Caspase9* after overexpression or silencing *ATG5* gene **(G,H,I,J)**.

Overexpression of *ATG5* inhibits cell apoptosis, promotes cell viability, whereas silencing *ATG5* produces opposite results. The results of CCK-8 assay for cell viability showed that the cells count in the ATG5-OE group was higher than that in ATG5-PC group at both 48 h and 72 h post-transfection (*p* < 0.05) (Figure 6A), accompanied by significantly increased expression levels of the proliferation-related genes *Bcl2* and *PCNA* (*p* < 0.01; Figures 6G,H). Additionally, the cell count in the si-ATG5-477 group was significantly lower compared to the si-ATG5-NC group at the same time points (*p* < 0.01) (Figure 6D), along with significantly decreased expression of *Bcl2* and *PCNA* (*p* < 0.01; Figures 6G,H). The apoptosis rate in the ATG5-OE group was significantly lower than that in the ATG5-PC group (*p* < 0.01) (Figures 6B,C), along with significantly decreased expression of the apoptosis-related genes *Caspase3* and *Caspase9* (*p* < 0.01; Figures 6I,J). The apoptosis rate in the si-ATG5-477 group was higher than that in the si-ATG5-NC group (*p* < 0.05) (Figures 6E,F), along with significantly elevated expression of *Caspase3* and *Caspase9* (*p* < 0.01; Figures 6I,J).

### 3.7 qPCR detection of autophagy and testosterone synthesized gene expression

**Figure 7:**
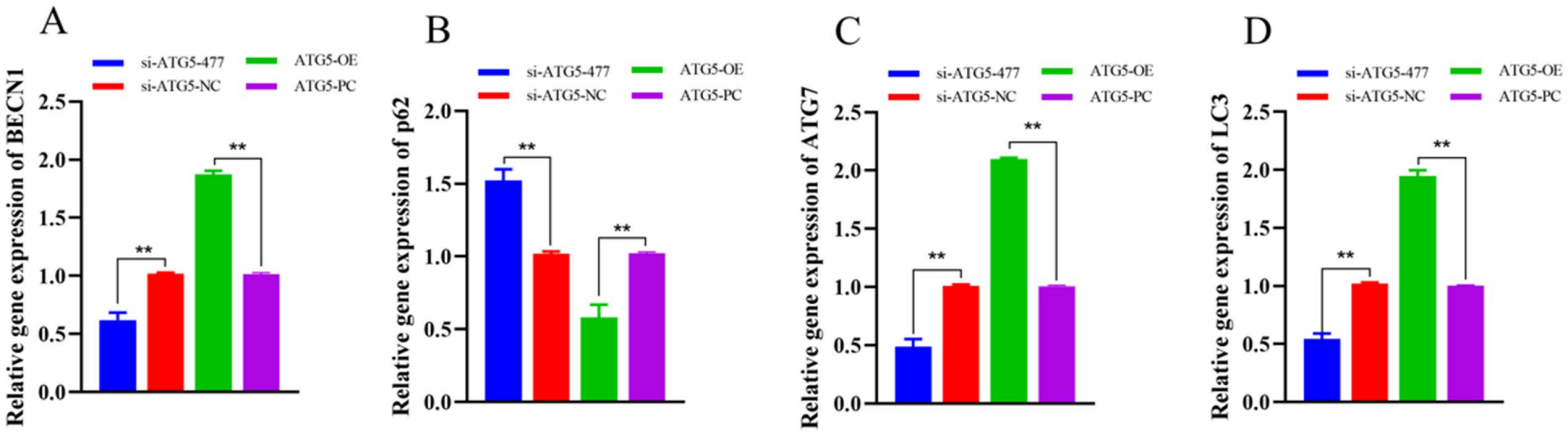
Effect of interference and overexpression of *ATG5* gene on *BECN1* **(A)**, *p62* **(B)**, *ATG7* **(C)**, and *LC3* **(D)** genes in Leydig cells of testicular of Hezuo pig.

qPCR analysis demonstrated that at 48 h post-transfection, the expression levels of autophagy-related genes (*BECN1*, *ATG7* and *LC3*) in the si-ATG5-477 group were significantly downregulated compared to the si-ATG5-NC group (*p* < 0.01), while *p62* expression was markedly upregulated (*p* < 0.01). Conversely, the ATG5-OE group showed significantly elevated expression of *BECN1*, *ATG7* and *LC3* (*p* < 0.01), accompanied by a significant reduction in *p62* expression (*p* < 0.01) compared to the empty vector control group. These findings indicate that *ATG5* overexpression enhances the expression of autophagy-related genes in Hezuo pig Leydig cells, while *ATG5* silencing suppresses this expression (Figure 7).

**Figure 8:**
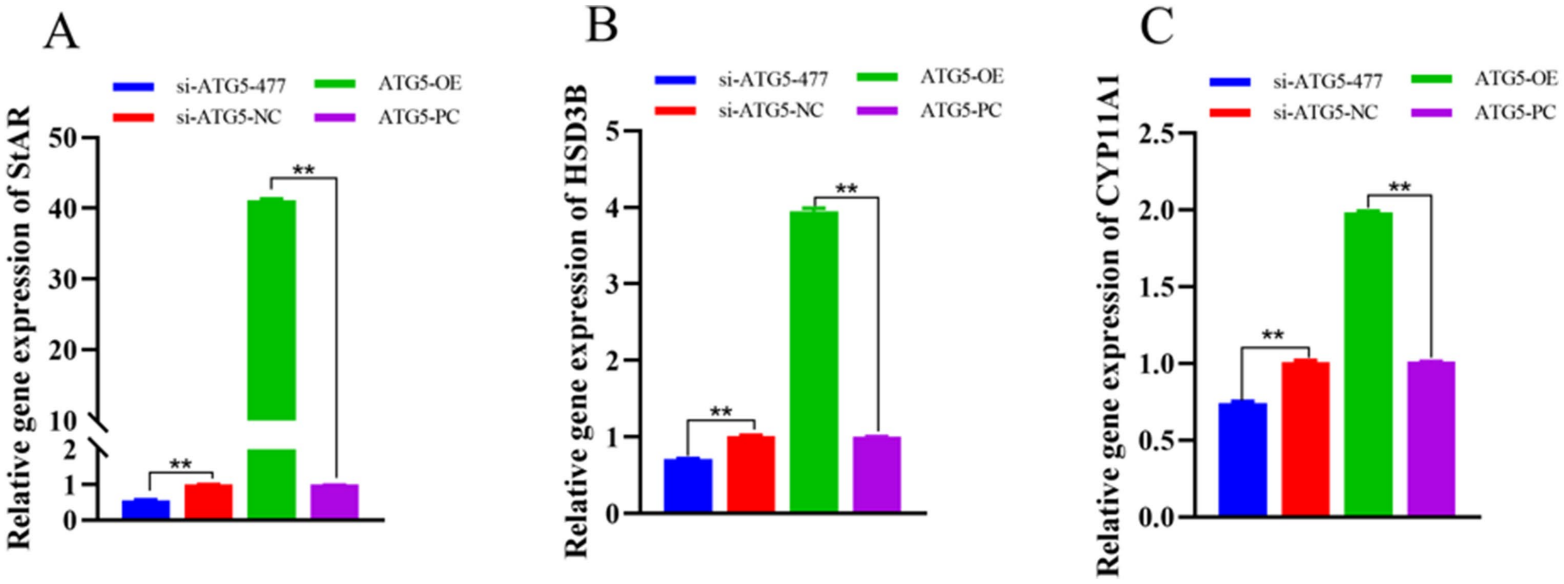
Effect of interference and overexpression of *ATG5* gene on *StAR* **(A)**, *HSD3B* **(B)**, and *CYP11A1* **(C)** genes in Leydig cells of testicular of Hezuo pig.

qPCR analysis revealed that at 48 h post-transfection, the expression levels of testosterone synthesis-related genes (*StAR*, *HSD3B* and *CYP11A1*) in the si-ATG5-477 group were significantly lower than those in the si-ATG5-NC group (*p* < 0.01). Conversely, the ATG5-OE group exhibited significantly higher expression levels of these steroidogenic genes compared to the empty vector control group (*p* < 0.01). These results indicate that *ATG5* overexpression upregulates the expression of testosterone production-related genes in Hezuo pig Leydig cells, while *ATG5* silencing downregulates their expression (Figure 8).

### 3.8 Western blotting detection of ATG5, StAR and LC3 protein expression

**Figure 9:**
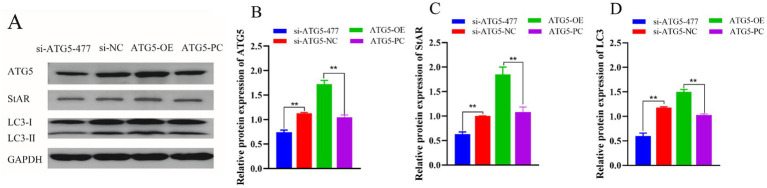
Effect of *ATG5* gene silencing or overexpression on expression of ATG5, StAR and LC3 protein of Hezuo pig Leydig cells. Western blot bands in Hezuo pig Leydig cells after silencing or overexpression *ATG5* gene **(A)**, Relative protein expression level of ATG5 in Hezuo pig Leydig cells after silencing or overexpression *ATG5* gene **(B)**, Relative protein expression level of StAR in Hezuo pig Leydig cells after silencing or overexpression *ATG5* gene **(C)**, Relative protein expression level of LC3 in Hezuo pig Leydig cells after silencing or overexpression *ATG5* gene **(D)**.

Western blotting was used to evaluate the expression levels of ATG5, StAR and LC3 protein. Results showed that compared to the control group, ATG5-OE group significantly increased the expression of ATG5, StAR and LC3 (*p* < 0.01), and si-ATG5-477 group could significantly reduce the expression of ATG5, StAR and LC3 (*p* < 0.01). Overexpression of *ATG5* promoted the expression of ATG5, StAR and LC3 proteins in Hezuo pig Leydig cells, whereas silencing of *ATG5* inhibited the expression of ATG5, StAR and LC3 proteins in Hezuo pig Leydig cells (Figure 9).

### 3.9 Effects of ATG5 gene silencing or overexpression on testosterone, autophagy gene BECN1, NPC1L1 and TSPO of Hezuo pig Leydig cells

**Figure 10:**
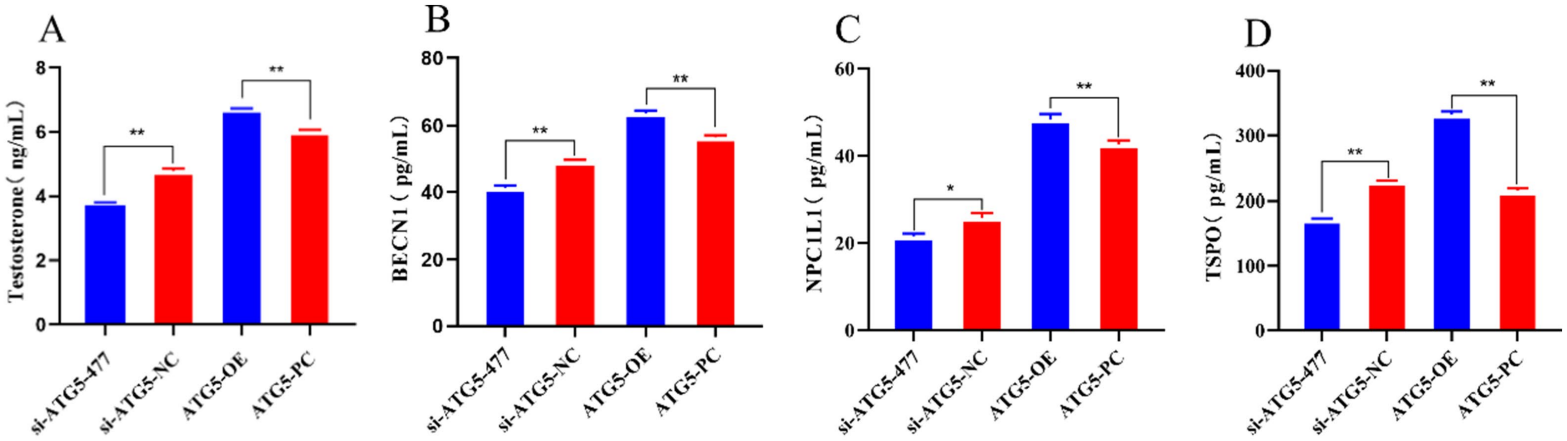
The content of testosterone, autophagy gene BECN1, NPC1L1, and TSPO after silencing and overexpression of *ATG5* gene.

Overexpression of *ATG5* increases the secretion testosterone, autophagy genes BECN1, NPC1L1 and TSPO secretion, while silencing of *ATG5* could decreases these levels. In this experiment, the levels of testosterone, autophagy genes BECN1, NPC1L1 and TSPO were quantified using ELISA 48 h post-transfection. The results showed that in the ATG5-OE group, the expression of testosterone, autophagy genes BECN1, NPC1L1 and TSPO of were significantly increased compared to the ATG5-PC group (*p* < 0.01). Conversely, in the si-ATG5-477 group, these levels were significantly lower than those in the si-ATG5-NC group (*p* values: testosterone < 0.01, BECN1 < 0.01, NPC1L1 < 0.05, and TSPO < 0.01) (Figure 10).

## 4 Discussion

### 4.1 Function of ATG5 gene and sequence characterization of ATG5 in Hezuo pig

In this study, we obtained the CDS region sequence of *ATG5* gene from Hezuo pig. Compared to the reference sequence, the CDS region of the Hezuo pig *ATG5* gene is 828 bp long, encoding 275 amino acids. We identified three nucleotide mutations, leading to two amino acid substitutions. These mutations may influence the function of testicular Leydig cells in Hezuo pigs by enhancing autophagy and facilitating the efficient transfer of cholesterol for testosterone and other steroid hormone synthesis, thereby ensuring normal reproductive performance.

The *ATG5* gene plays a crucial role in the early stages of autophagosome formation. Studies have shown that changes in ATG5 protein levels (whether increased or decreased), affect autophagy flux. Specifically, knocking down *ATG5* blocks autophagosome formation (14). ATG5 can form a complex with ATG12, an ubiquitin-like protein that is highly unstable in its free state and can be directly degraded by ubiquitination. The stability of ATG12 is significantly enhanced upon forming a complex with ATG5. Upon binding with ATG12, ATG5 can form the ATG12-ATG5-ATG16 complex with ATG16, facilitating the elongation of autophagic vesicles. In this process, ATG5 acts as a coupling switch. Additionally, the ATG12-ATG5 complex accelerates the lipidation of ATG8 (LC3), promoting the formation of an E3-like enzyme activity required for autophagy (16, 17). The ATG5 complex binds to the autophagic vesicle membrane and promote the aggregation of LC3 (ATG8) to autophagic vesicles (16). Currently, no studies on the *ATG5* gene in Hezuo pig have been reported. Han et al. (40) found that the CDS region of the chicken *ATG5* gene is 831 bp long and encode 275 amino acids. Lei et al. (41) report that 828 bp CDS sequence of buffalo *ATG5* gene encodes a protein of 275 amino acids, consistent with our findings.

### 4.2 Effect of ATG5 on proliferation and apoptosis of Leydig cells of Hezuo pig

In this study, we found that overexpression of *ATG5* promoted the proliferation and inhibited the apoptosis of testicular Leydig cells in Hezuo pig, while silencing of *ATG5* suppressed proliferation and enhanced apoptosis in these cells. There is a close relationship between cellular autophagy and apoptosis. When organisms experience infection, nutritional deficiency, stress, or radiation, they initiate cellular autophagy as a self-protective mechanism. However, if external stimuli persist, self-protection mechanisms fail, leading to the activation of apoptosis. It has been proposed that autophagy may serve as an apoptotic pathway. Consequently, autophagy is often referred to as type II apoptosis, which induces cell death by degrading essential cellular components (e.g., mitochondria), thereby depriving the cell of the energy necessary for survival (42–44). From a molecular perspective, several genes are shared between autophagy and apoptosis, including *ATG5*, *Bcl-2*, *p53* and *ARF*. These genes play crucial regulatory roles in both processes, and their activation or silencing influences both pathways. In apoptotic cells, *ATG5* is cleaved by calpain, resulting in the translocation of its N-terminal fragment to mitochondria. This fragment mediates the release of cytochrome C by interacting with the pro-survival Bcl-2 family member Bcl-xL (45). Non-conjugated ATG12 binds to Bcl-2 family proteins and promotes apoptosis (46). Beclin-1 is an essential regulator of autophagosome formation and also plays a role in regulating apoptosis. As a BH3-only protein in the Bcl-2 family, Beclin-1 interacts with Bcl-2 localized in the endoplasmic reticulum via its BH3 domain, thereby inhibiting autophagy (47).

### 4.3 Effect of ATG5 on testosterone production by Leydig cells of Hezuo pig

The results of this study indicated that overexpression of *ATG5* significantly increased the expression levels of ATG5, StAR, and LC3 protein, autophagy and testosterone synthesized gene Expression, as well as the contents of testosterone, BECN1, NPC1L1, and TSPO in the testicular Leydig cells of Hezuo pigs. This suggests that *ATG5* regulates testosterone production via cellular autophagy, potentially through the modulation of StAR, NPC1L1, and TSPO.

Testosterone is a crucial sex hormone in mammals, playing a key role in reproductive functions including spermatogenesis and the maintenance of secondary sexual characteristics. In male mammals, testicular Leydig cells are the main site of testosterone synthesis. Exposure to environmental stimuli, such as toxins (48) or hypoxia (49), can affect cellular autophagy and alter testosterone secretion. Chen et al. (5) observed that both the steroidogenic activity and ultrastructural features of testicular Leydig cells in dairy goats change with age, based on ex vivo and *in vivo* experiments. Compared to juveniles, Leydig cells in sexually mature and adult goats contain numerous smooth endoplasmic reticula, mitochondria, and lipid droplets, which form the foundation for testosterone synthesis. Moreover, Leydig cells from sexually mature and adult goats exhibit higher autophagic activity compared to juveniles, indicating that autophagy contributes to testosterone synthesis primarily by degrading mitochondria and endoplasmic reticula in these cells.

Some researchers fed mice a zinc-deficient diet for 8 weeks. The results indicated that compared to the normal diet group, the zinc-deficient diet led to testicular structural abnormalities and impaired autophagy, with significant reductions in *ATG5* and Beclin1 expression, as well as a notable decrease in testosterone levels (50). Xiao et al. (51), collected ovarian granulosa cells from patients with polycystic ovary syndrome (PCOS), a common gynecological endocrine disorder characterized by hyperandrogenism, and from patients without PCOS. RT-PCR analysis showed that the mRNA expression of autophagy-related genes *ATG5*, *ATG7*, and *BECN1* was significantly elevated in the ovarian granulosa cells of PCOS patients. Esmaeilian et al. (52), isolated testicular tissues from males undergoing orchiectomy and either silenced autophagy genes (*Beclin1* and *ATG5*) using siRNA and shRNA or altered autophagy via pharmacological inhibition. Results showed that both approaches significantly decreased the production of testosterone (T), progesterone (P), and estradiol (E2) in isolated testicular tissues. This confirms that the human testis produces steroid hormones including testosterone, estrogen, and progesterone through an autophagy-mediated pathway. Yang et al. (53), observed that autophagy was significantly reduced in the testicular interstitial cells of non-breeding male naked mole-rats (NMRs) compared to breeding NMRs, accompanied by significant decreases in *ATG7*, *ATG5* expression, autophagosome count, and declines in *StAR* and testosterone production. This reduction correlated with decreased autophagic activity. Li et al. (54), reported that reduced testosterone levels were linked to decreased autophagic activity in aged rat Leydig cells. Furthermore, knockdown of *Beclin1* resulted in autophagic deficiency, leading to decreased StAR protein expression and testosterone production. Gong et al. (55), found that enhancing autophagy in porcine Leydig cells increased testosterone levels and StAR protein expression.

TSPO is a drug-and cholesterol-binding protein that is particularly abundant in steroid synthesizing cells (35). Previous studies have demonstrated that TSPO levels decrease in senescent Leydig cells (LCs), and this reduction correlates with lower circulating testosterone levels in aged rats (56). The testosterone-producing activity of Leydig cells is diminished when TSPO expression is entirely absent. TSPO deficiency results in the disruption of mitochondrial function and membrane dynamics. Enhancing mitochondrial fusion might offer a therapeutic approach for maintaining or restoring testosterone levels. The TSPO agonist FGIN-1-27 stimulates testosterone production in Leydig cells, leading to increased serum and intratesticular testosterone levels (34, 57). Recent studies indicate that NPC1L1 is a transmembrane cholesterol transporter crucial for cholesterol uptake, lipid homeostasis regulation, and providing substrates for steroid hormone synthesis. Cholesterol depletion has been linked to autophagy (36, 37).

## 5 Conclusion

*ATG5* in Hezuo pigs showed high expression at 4 months of age, particularly in testicular and lung tissues. Silencing and overexpression vectors for the *ATG5* gene were constructed, which effectively reduced or increased *ATG5* gene expression in Leydig cells. Overexpression of *ATG5* was observed to inhibit apoptosis, enhance cell viability, elevate the expression levels of ATG5, StAR and LC3 proteins, and increase testosterone secretion as well as the expression of autophagy-related genes (*BECN1*, *ATG7* and *LC3*). In contrast, silencing *ATG5* produced opposite effects. These findings suggest that manipulating *ATG5* expression-either through interference or overexpression-can regulate testosterone synthesis, potentially via autophagy-mediated cholesterol transport regulation. These findings lay a scientific foundation for investigating testosterone production and reproductive disorders not only in Hezuo pigs but also in other male mammals.

## Data Availability

The original contributions presented in the study are included in the article/supplementary material, further inquiries can be directed to the corresponding author.
